# Composites of Platinum-Iridium Alloy Nanoparticles and Graphene Oxide for the Dimethyl Amine Borane (DMAB) dehydrogenation at ambient conditions: An Experimental and Density Functional Theory Study

**DOI:** 10.1038/s41598-019-52038-3

**Published:** 2019-10-29

**Authors:** Betül Sen, Ayşenur Aygun, Aysun Şavk, Mehmet Harbi Çalımlı, Mehmet Ferdi Fellah, Fatih Sen

**Affiliations:** 1 Sen Research Group Biochemistry Department, Faculty of Arts and Science, Dumlupınar University, Evliya Çelebi Campus, 43100 Kütahya, Turkey; 2 Tuzluca Vocational High School, Igdir University, Igdir, Turkey; 3 Department of Chemical Engineering, Bursa Technical University, Mimar Sinan Campus, 16310 Bursa, Turkey

**Keywords:** Chemistry, Catalysis, Heterogeneous catalysis

## Abstract

In this paper, we present the synthesis, characterization, catalytic and computational studies of Composites of Platinum-Iridium Alloy Nanoparticles and Graphene Oxide (PtIr@GO) for dimethylamine borane (DMAB) dehydrogenation. The prepared PtIr@GO nanocatalysts were synthesized using an ethanol super-hydride method, and the characterization procedures for PtIr@GO alloy nanoparticles were carried out by various advanced spectroscopic methods like X-ray Diffraction (XRD), X-ray photoelectron spectroscopy (XPS), Transmission Electron Microscopy(TEM) and high-resolution transmission electron microscopy (HRTEM). Additionally, catalytic activity, reusability, substrate concentration, and catalyst concentration experiments were performed for DMAB dehydrogenation catalyzed by PtIr@GO alloy nanomaterials. According to the results obtained in this study, PtIr@GO NPs catalyst was found to be active and reusable for the DMAB even at ambient conditions. Besides, DFT-B3LYP calculations have been utilized on PtIr@GO cluster to reveal the prepared catalyst activity. The calculated findings based on DFT was found to be a good agreement with experimental results.

## Introduction

**Figure 1:**
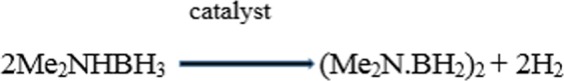
Hydrogen release reaction of DMAB using an appropriate catalyst.

The using of fossil fuel resources with many drawbacks have forced people predicting the future energy scenario of the world. In this regard, finding alternative energy sources for a sustainable environment is became a necessity. Currently, hydrogen is paid attention as a clean, renewable, environmentally friendly energy source. However, synthesis materials having high catalytic activity, safe and storage capacity is an unresolved problem^1^. Hydrogen present in the structure of various sources, one of them ammonia borane, ammonia borane derivatives are preferred because of their high stability, hydrogen content, easiness of using and non-flammable properties^2,3^. Dimethylamine borane (DMAB) is an ammonia borane derivative and it has taken an extensive interest in the usage of hydrogen storage studies^4^. However, the ammonia borane derivatives should be used in combination with suitable catalysts. Hitherto, several studies^5,6^ have been conducted about the catalytic activity of ammonia borane and hydrogen release from their derivatives to find a proper solution for this issue. Hydrogen release in the result of the reaction of dimethylamine borane (CH_3_)_2_NHBH_3_, DMAB) reaction in the existence of a suitable catalyst has been achieved, and 3.5% by weight of hydrogen was obtained^7–15^. Although homogeneous catalysts^16^ showed the best activity, heterogeneous catalysts have been used widely in catalytic reactions, because they have some superiors such as stability, activity, recycle and having high catalytic lifetime^17–21^. The mentioned catalysts were prepared with their process and characterized various methods like using Raman, XPS, XRD, HRTEM, and TEM. However, so far, a catalyst having a sufficient catalytic activity and reusability has not been produced. Pt-based nanoparticles are important nanoparticles that have been used in the conversion of many inorganic and organic materials^22–24^. So far, we have conducted many studies on catalysts and about their catalytic studies, and it has been seen that platinum and graphene oxide-based catalysts are very important and active in these studies. Therefore, in this study, it is aimed to create a new nanocatalyst with graphene oxide support to combine the effects of iridium with platinum and to increase the efficiency of platinum. For this purpose, for the first time, the synthesis, characterization and kinetic activity of platinum and iridium supported graphene oxide (PtIr@GO) alloy nanoparticles were investigated for DMAB catalytic dehydrogenation. The catalytic reaction of DMAB is shown as shown below (Fig. 1).

The preparation of PtIr@GO alloy nanoparticles was performed by reducing PtCl_4_ and IrCl_3_ in THF, in the presence of ethanol - super hydride and graphene oxide at room conditions. After the preparation of nanoparticles, PtIr@GO were characterized using some spectroscopic analysis (TEM, HRTEM, XRD, XPS, Raman, etc) and it has shown that the structure of Pt and Ir nanoparticles were highly crystalline and well decorated on graphene oxide. After that, PtIr@GO alloy nanoparticles was carried out for the dehydrocoupling of DMAB at room conditions. Thanks to this study, it was shown that PtIr@GO alloy nanoparticles could be used as an effective catalyst for catalytic dehydrogenation reaction of DMAB. Besides, DFT-B3LYP calculations have been utilized on PtIr@GO nanoparticles to reveal the catalytic activity of PtIr@GO in DMAB reaction.

## Experimental

### The preparation of bimetallic PtIr@GO alloy nanoparticles

Composites of Platinum-Iridium Alloy Nanoparticles and Graphene Oxide have been synthesized by super hydride-ethanol reduction method. For this purpose, a mixture containing PtCl_4_ and IrCl_3_ compounds (0.25 mmol) and graphene oxide (25 mg/mL) were prepared and the reduction of Pt (IV) and Ir (III) to Pt (0) and Ir (0), respectively were performed using super hydride and ethanol according to the chemical reduction method^25^. The changing color of the resulting mixture to brown color indicated the formation of Pt (0) and Ir (0) on the surface of GO support and then the obtained solid sample was dried under an inert atmosphere at an oven.

### Observation catalytic activity of PtIr@GO alloy nanoparticles in DMAB hydrogen release reaction

The catalytic reactions of the dehydrogenation of DMAB on PtIr@GO alloy nanoparticles were carried out in a cylindrical glass vessel connecting a scaled cylinder glass tube containing water as shown in Fig. S1. Before starting the experiment, the oxygen in the reaction medium was removed and then the nitrogen was sent to the reaction medium. These preparations ensure that the reaction medium was to be inert. All experiments were carried out at the same conditions. 1 mmol DMAB (60.70 mg) was added to 4 mL THF, mixed and this mixture was transferred into the reaction vessel. It was waited for 15 minutes to reach thermal equilibrium. The reaction was initiated by transferring the appropriate amount of catalyst and containing 1 mL of THF to the reaction vessel at the thermally equilibrated condition. The amount of hydrogen gas released was recorded by displacement of the water in the cylindrical container. The completion of the reaction and hydrogen release was understood upon the water level keeping constant.

### Computational method

The theoretical calculations were performed using Density Functional Theory (DFT)^26^ in this study. In theoretical works about catalytic studies of the prepared catalyst, the Gaussian 09^27^ software operate with B3LYP-Hybrid formalism process was used^28,29^. It has been known that one of the DFT methods for the high-quality procedure of theoretical calculations for organic chemistry is the B3LYP method. The LanL2DZ basis set was used in calculations for Pt and Ir metals. In theoretical works, the fasis set about the calculation of atoms like O, H, and C present in the cluster is 6–31 G(d,p).

The graphene oxide (GO) cluster used for calculations has 40 C atoms and 10 Oxygen atoms meaning there are 10 epoxy groups. The structure of GO used is represented in Fig. S2 in Supporting Information. H atoms are required to saturate the dangling bonds of the C atoms for neutralizing the cluster used in the study. The similar structure of GO matrix used in this study has been used in a recent theoretical study^30^. One Pt and one Ir atom have been used to obtain PtIr@GO cluster that is to represent the PtIr@GO catalyst. These metal atoms have been located on p^1^ and p^2^ points on the GO cluster. A similar strategy in order to obtain Pt-decorated GO cluster has been utilized during DFT calculations in a study conducted about platinum decorated on GO for methane/methanol^30^. Two Pt atoms have been located on some points that are similar to the points used in that study^30^. In present work, atoms were entirely kept free.

Balance geometry calculations have been used to optimize geometries in adsorption energy studies. Additionally, zero-point energy (ZPE) and energy difference findings were also investigated. These values were computed by using the freq keyword in Single Point Energy (SPE) calculation. In addition, in Gaussian software vibrational frequency, thermal energy, enthalpy, and free energy data have been obtained by SPE calculations at 298 K and 1 atm pressure^31^. The calculated theoretical energy values were found as given below:

1 $${\rm{E}}={{\rm{E}}}_{{\rm{electronic}}}+{\rm{ZPE}}+{E}_{{\rm{vibrational}}}+{E}_{{\rm{rotational}}}+{E}_{{\rm{translational}}}$$

2 $${\rm{H}}={\rm{E}}+\mathrm{RT}\,$$

3 $${\rm{G}}={\rm{H}}-{\rm{TS}}$$

In these equations; H, E, G, T and S are enthalpy, thermal energy, free energy, temperature (K) and entropy in vibrational frequency data, respectively. HOMO geometric representation and LUMO geometric representation, and HOMO/LUMO energies s were found by using the population analysis. In order to have some info about the activity of cluster the values of chemical hardness and potential, the electronegativity and the electrophilicity were achieved. Here ϵ_LUMO_ is defined as the lowest unoccupied molecular orbital energy and ϵ_HOMO_ is defined as the highest occupied molecular orbital energy. The equations have been based on the method of Koopman^32–35^ and given as follows.

4 $${\rm{Chemical}}\,{\rm{hardness}}\,{\rm{value}}\,({\rm{\eta }})=\frac{I-A}{2}$$

5 $${\rm{Chemical}}\,{\rm{potential}}\,{\rm{value}}\,(\mu )=-\,\frac{I+A}{2}$$

6 $${\rm{Electronegativity}}\,{\rm{value}}\,({\rm{\lambda }})=-\,\mu $$

7 $${\rm{Electrophilicity}}\,{\rm{value}}\,({\rm{\omega }})=\frac{{\mu }^{2}}{2\eta }$$

Here, $$I\cong -\,{{\epsilon }}_{HOMO}\,and\,A\cong -\,{{\epsilon }}_{LUMO}$$

The theoretical methodology utilized here is given in Supporting Information. Multiwfn software is used to obtain the map’s distribution of electron localization functions (ELF) and the electron density (ED)^36^. Moreover, atoms charges are known as Mulliken was found using Mulliken population calculations^37^.

## Results and Discussion

Composites of Platinum-Iridium Alloy Nanoparticles and Graphene Oxide were easily prepared by the reducing of platinum (IV) and iridium (III) chlorine salts in a THF solution with the super hydride ethanol reduction method at ambient conditions. Upon reduction of Pt (IV) and Ir (III) to Pt (0) and Ir (0), respectively, the precipitation and agglomeration were formed without graphene oxide. Furthermore, the stability of the synthesized catalyst was tested and found to be stable. This stable structure is thought to be related to GO which is used as a support material in the catalyst. TEM, HRTEM, Raman, XRD, and XPS analysis were used for the morphological and structural distributions of PtIr@GO catalyst.

**Figure 2:**
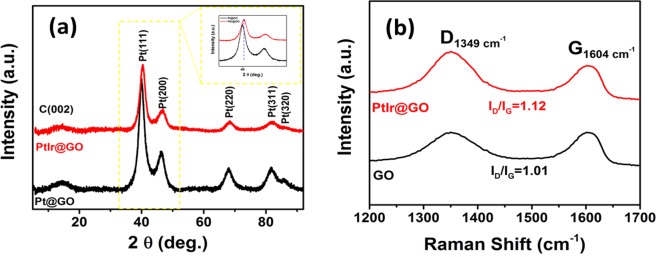
(**a)** Powder X-ray diffraction pattern of PtIr@GO nanoparticles in the 2θ range of 0–95° **(b)** Raman spectrum of GO and PtIr@GO.

The result of the XRD analysis is shown in Fig. 2a. The diffraction lines correspond to the peaks (111), (200), (220), (311), and (320) planes at about 2θ = 39.95°, 46.60°, 67.50°, 81.20° and 86.70°, and these values are platinum-iridium nanoparticles show that the face-centered cubic (fcc) crystal lattice structure^25,38,39^. As shown in Fig. 2a, the diffraction pattern of PtIr@GO slightly shifted compared to the Pt@GO nanoparticles due to the alloy formation in prepared nanocomposites. Using the following equation, the lattice parameter of PtIr@GO was calculated as 3,922 Å.

$$Sin\,\theta =\frac{{\boldsymbol{\lambda }}\sqrt{{{\boldsymbol{h}}}^{{\bf{2}}}+{{\boldsymbol{k}}}^{{\bf{2}}}+{{\boldsymbol{l}}}^{{\bf{2}}}}}{{\bf{2}}{\boldsymbol{a}}}$$

Further, the mean PtIr@GO particle size was calculated using the full width at half maximum of peaks in the P-XRD analyses (Fig. 2(a)) with the following Scherrer equation^40^.

$$d(\AA )=\frac{{\boldsymbol{k}}{\boldsymbol{\lambda }}}{{\boldsymbol{\beta }}\,{\bf{\cos }}\,{\boldsymbol{\theta }}}$$

Raman spectrochemical analysis was carried out for further details about the chemical compositions and morphological changes for Pt@GO and PtIr@GO nanoparticles. Because Raman spectroscopy is a very suitable technique for this purpose^41,42^ Fig. 2b shows the results of the Raman analysis. Two distinct peaks present in Fig. 2b correspond to the 1349 cm^−1^ and 1604 cm^−1^ ranges, and these peaks were assigned to the D band and the G band. The change in the D band is explained by the presence of PtIr nanoparticles in the graphene oxide material, which also indicates the formation of the composites of Platinum, iridium and graphene oxide.

**Figure 3:**
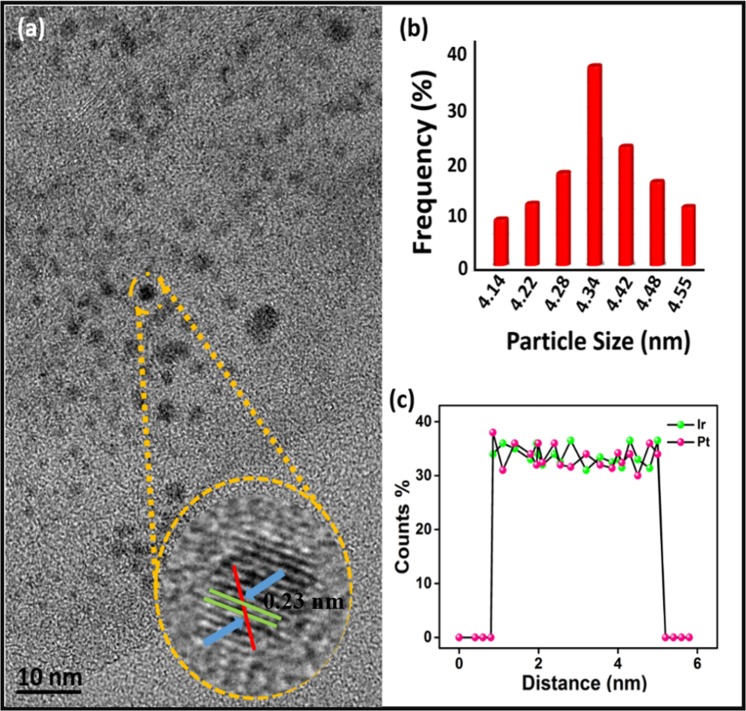
(**a)** TEM analyses patterns **(b)** particle size histogram **(c)** The EELS analyses of PtIr@GO nanoparticles.

In addition, the platinum-iridium metals distributions on GO support materials and the average of PtIr@GO nanoparticles were investigated by TEM and HRTEM patterns. In order to count the average particle size of PtIr@GO alloy nanoparticles, about 100 particles present in TEM pattern (Fig. 3a) were counted and accordingly the average of particle size of the catalyst was calculated as 4.14–4.55 nm (Fig. 3a,b). The average particle size was calculated as 4.34 ± 0.36 nm according to the TEM results. In the HRTEM analysis, the atomic lattice fringe portion of PtIr@GO nanoparticles was observed as 0.23 nm. Furthermore, in Fig. 3c, TEM-electron energy loss spectroscopy (EELS) analysis showed that the PtIr metals in the PtIr@GO alloy were in a 1:1 ratio.

**Figure 4:**
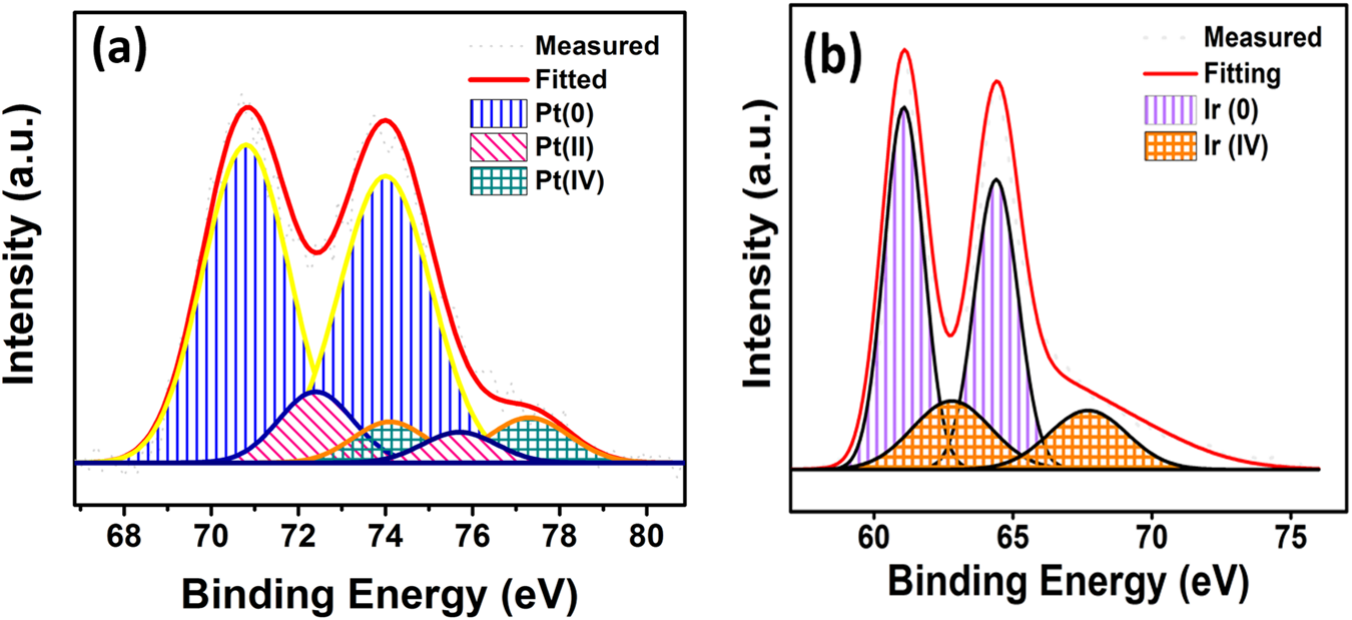
The images of XPS analysis for **(a)** Pt 4f, **(b)** Ir 4f region in prepared PtIr@GO nanoparticles.

Further, XPS analyses were performed to illuminate the electronic and oxidation status of palladium and iridium metals on GO supporting materials. 4f spectrum fields of Pt and Ir metals were examined by applying Gaussian Lorentzian technique. The densities of the species in the nanomaterials were calculated by counting by flattening the background in the graphs obtained from XPS analysis. The binding energy of C1s (Fig. S3) was performed as a standard value to investigate the binding energies found in XPS analyses. The binding energy of about 71.0 eV, corresponding to Pt 4f_5/2_, as shown in Fig. 4a, indicating that there is mostly metallic platinum on the catalyst surface^43,44^. Some oxidized Pt patterns of Pt (II) and Pt (IV) were formed and these oxidized patterns may have probably been because of oxidized or unreduced nanoparticle samples. In Fig. 4b, when Ir 4f peak is examined, it’s seen that there is mostly metallic Iridium and weakly Ir (IV) signals. This shows that platinum and iridium metals are mostly co-existed on the surface of the catalyst, so it could be concluded that Pt and Ir nanoparticles form alloy in the presence of graphene oxide.

After full characterization of the prepared Composites of Platinum-Iridium Alloy Nanoparticles and Graphene Oxide, the hydrogen production was determined for the catalytic activity PtIr@GO alloy nanoparticles in the dehydrogenation of DMAB under mild conditions. By starting the catalytic reaction of DMAB within an appropriate amount of the PtIr@GO alloy catalyst in THF solution, and the releasing hydrogen gas started without observing any induction time. The turnover frequency value was calculated as 225.64 h^−1^. When the same catalytic reaction was repeated with Pt (IV) and Ir (III) chlorine salts as pre-catalyst, it was observed seven minutes induction time and a 10–15% conversion of DMAB (Fig. S4). Because only chloride anion present as a stabilizer in the solution environment, and platinum-iridium metal nanoparticles were aggregated and precipitated within 10 minutes^45^. This result shows the importance of the stabilization effect of GO on the catalyst.

### Evaluation of some activation parameters of PtIr@GO alloy catalyst for the DMAB dehydrogenation reaction

**Figure 5:**
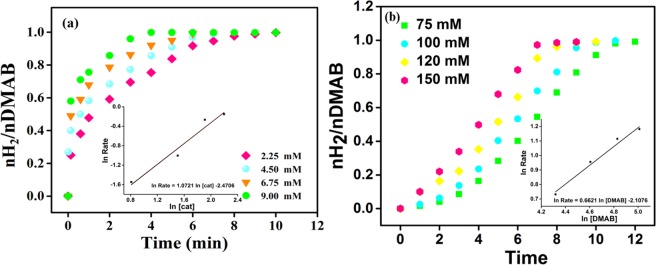
(**a)** The graph of hydrogen release with different amounts of PtIr@GO in a range of 2.25–9.00 mM and **(b)** with different amounts of DMAB under mild conditions.

In order to evaluate the kinetic parameters of PtIr@GO NPs in DMAB catalytic reaction; temperature, catalyst concentration, substrate concentration, and reuse experiments were carried out. In order to estimate the effect of catalyst concentration on the catalytic reaction of DMAB some experiments containing different PtIr@GO catalysts concentration in a range of 2.25–9.0 mM) were performed under room conditions, and their results are seen in Fig. 5a. NMR results of DMAB demonstrated the completion catalytic reaction (DMAB (δ = ~12.8) to metal borate (δ = ~4.9 ppm))^46,47^. A plot containing hydrogen evaluation versus to time for different PtIr@GO catalysts concentrations for DMAB at mild conditions can be seen in Fig. 5a. As seen the catalytic reaction of DMAB, containing PtIr@GO catalysts was initiated without observing any induction time. The hydrogen evaluation was recorded, and a linear graph was obtained as given inside of Fig. 5a. The slope of a plot inside of Fig. 5a was calculated as 1.0721 that is related to the first order with respect to the PtIr@GO concentration. In order to evaluate the effect of DMAB amount on catalytic efficiency some experiments containing different DMAB amount in the range of 75–150 mM were carried out.

The experimental findings performed using different DMAB amount are represented in Fig. 5b. As shown in this figure, a linear plot having slopes of 0.6621 was obtained. In the light of these results, the equation of the catalytic dehydrogenation rate of DMAB including PtIr@GO nanoparticles was attained as follows;

$$-\frac{d[{(C{H}_{3})}^{2}HNB{H}_{3}]}{dt}=+\,\frac{d[{({(C{H}_{3})}^{2}NB{H}^{2})}^{2}]}{dt}=+\,\frac{[{H}^{2}]}{dt}={[\mathrm{PtIr}@\mathrm{GO}]}^{1.0721}{[{\rm{DMAB}}]}^{0.6621}$$

**Figure 6:**
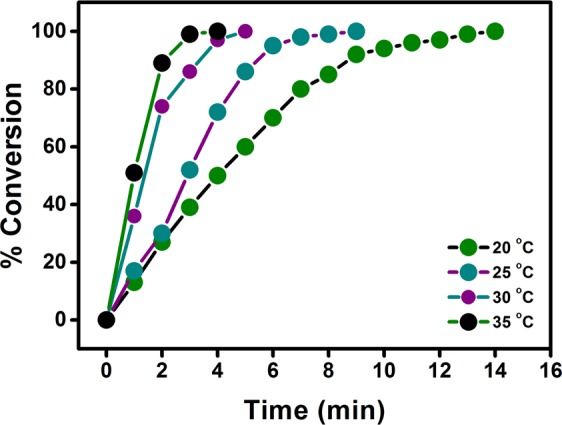
Graph of catalytic conversion of DMAB with PtIr@GO (7.5% mol) at different temperatures.

**Figure 7:**
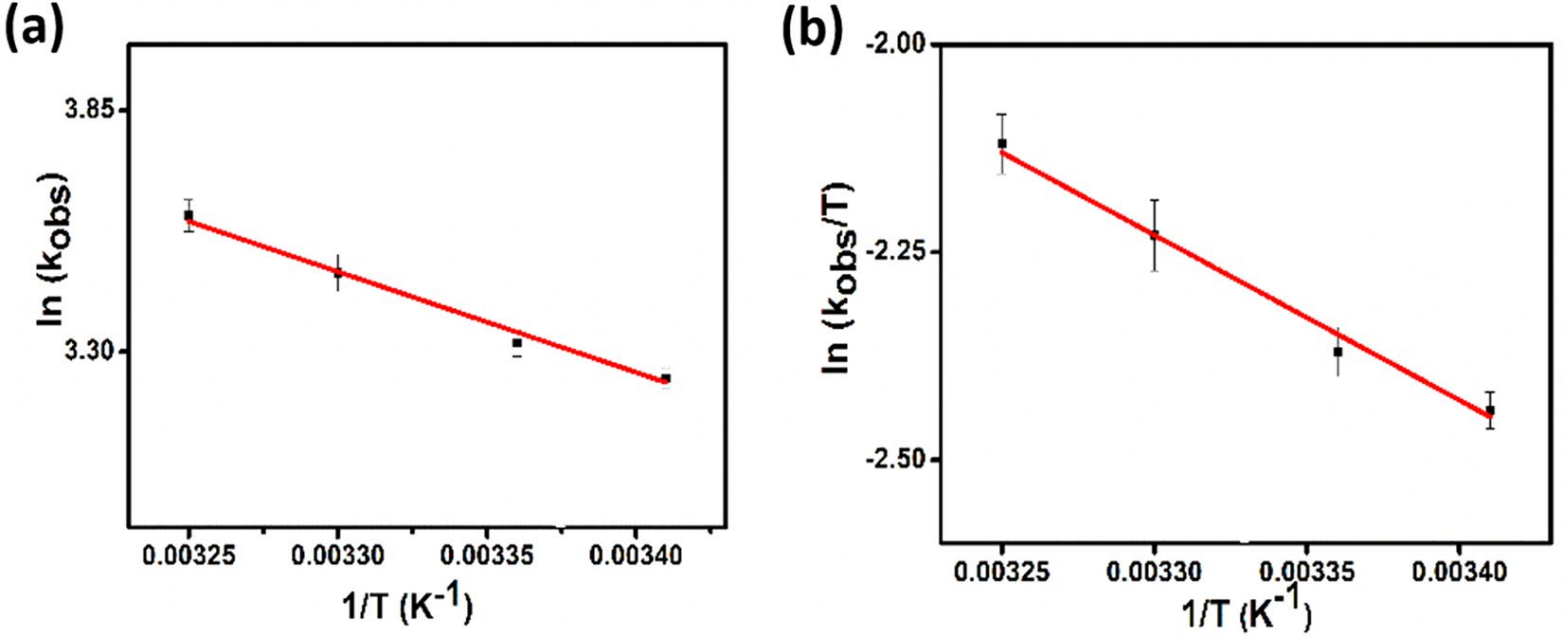
(**a**) Arrhenius and **(b)** Eyring plots for dehydrogenation reaction of DMAB containing PtIr@GO.

Figure 6 shows the conversion of DMAB catalyzed by PtIr@GO nanoparticles at different temperatures. It can be seen in experimental results, the reaction rate is increased with increased temperature. As shown in the graph, the conversion rate increased linearly in all increased temperatures and no induction time was observed. The ratios of hydrogen gas released were calculated for 20, 25, 30 and 35 °C. The rate dehydrogenation constants of DMAB reaction containing PtIr@GO were calculated from Fig. 7 for different temperatures. The activation parameters such as activation energy (Ea: 19.06 ± 2 kJ mol^−1^), entropy (ΔS: −178.18 ± 2 J mol^−1^ K^−1^) and enthalpy (ΔH: 16.53 ± 2 kJ mol^−1^) were obtained using Arrhenius and Eyring equations and were presented in Fig. 7a,b, respectively^48,49^.

Besides, the low and negative value of entropy change (ΔS: −178.18 ± 2 J mol^−1^ K^−1^) of DMAB reaction catalyzed PtIr@GO alloy nanoparticles indicates the dissociative mechanism.

### The reusability tests of PtIr@GO alloy nanoparticles in DMAB catalytic reactions

For the reusability experiments, the prepared PtIr@GO catalyst was kept under vacuum and in dry medium to maintain the stability of the nanoparticles. The catalytic activity and reusability experiment were carried out for fourth runs. The PtIr@GO alloy nanoparticles maintained its starting catalytic activity up to 77% at ambient conditions. The catalytic activity and reusability of PtIr@GO alloy nanoparticles for DMAB catalytic reaction can be shown in Fig. S5. The small amount of agglomeration of the nanoparticles was observed towards the end of the catalytic reaction, and a 23% decrease in catalytic activity may be associated with this agglomeration (Fig. S6). Besides, the comparison of the TOF values of PtIr@GO nanoparticles is given in Table S1. It can be seen in the table, the catalyst (PtIr@GO) used in this study exhibited the best TOF compared the heterogeneous catalysts used for DMAB dehydrocoupling reaction. The surface area of prepared PtIr@GO was also found to be 255 m^2^/g with the help of nitrogen adsorption-desorption technique.

**Figure 8:**
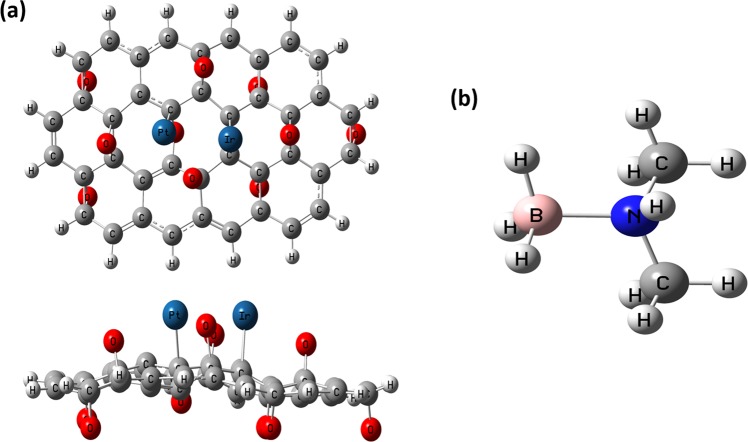
Optimized geometries: **(a)** PtIr@GO cluster with top view and side view, **(b)** DMAB molecule.

The experimental findings can be explained the ultrafine structure, high % metal content of PtIr@GO NPs, the synergistic effect of Pt, Ir and GO, highly stabilized GO with the metal alloys. The comparisons of results found from catalysts tested and present in the literature for DMAB with the results of this study revealed that Composites of Platinum-Iridium Alloy Nanoparticles and Graphene Oxide were determined as a highly active, and stable catalyst for hydrogen evolution from the DMAB. These results can be explained with probable reasons as (i) reaching a stable state of GO (ii) forming an optimum size particle of PtIr@GO and (ii) synergy of Pt and Ir in the catalyst structure. Besides, explain the high activity of PtIr@GO catalyst, some of the theoretical calculations were performed. For this purpose, firstly EG calculation was used to optimize the GO cluster. The optimized structure of GO cluster is represented in Fig. S2 in Supporting Information. Then PtIr@GO cluster was evaluated by employing EG studies. Neutral charge and the sextet SM have been used for this optimization of PtIr@GO cluster. The sextet SM corresponds to the lowest SPE energy of PtIr@GO composite. And again, sextet SM has been evaluated for the contents of the PtIr@GO catalysts for DMAB. Two possibilities for the adsorption of Pt and Ir atoms on the GO cluster have been utilized for EG calculations. These are the location of Pt-Ir atoms on p1-p2 sites respectively and the location of Ir-Pt atoms on p1-p2 sites respectively. Total energy values (including ZPE correction) for optimized PtIr@GO clusters have been computed as −2508.774611 and −2508.765568 a.u. for these clusters, respectively. This indicates that the location of Pt-Ir atoms on p^1^-p^2^ sites respectively is the most favorable sites for the location of Pt and Ir atoms on GO cluster. Figure 8 gives EGs for PtIr@GO cluster. SM and Charge for DMAB molecule (as adsorbing molecule) were found to be singlet and neutral, respectively. The geometry for DMAB structure optimized is given in Fig. 8b.

**Figure 9:**
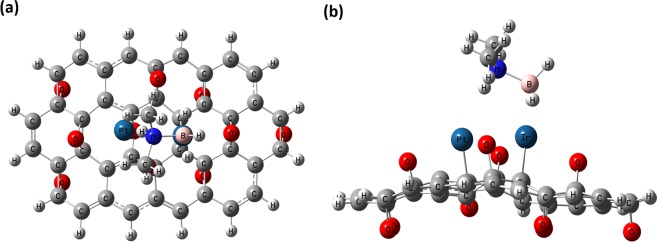
Optimized geometry for adsorbed DMAB molecule on PtIr@GO cluster: (**a**) Top view and (**b**) Side view.

With the determined optimized geometries of the obtained nanoparticle and moleculer structure of DMAB, adsorption of DMAB was examined on PtIr@GO cluster by EG calculations. Two possibilities (on Pt atom and Ir atom) for the adsorption of DMAB on PtIr@GO cluster have been utilized for EG calculations. Total energy values (including ZPE correction) for adsorption of DMAB on PtIr@GO cluster have been calculated to be −2670.547735 and −2670.498503 a.u. for these possibilities respectively, which designates that adsorption of DMAB on Ir atom on p^2^ site is most favorable. Optimized geometry for adsorbed DMAB molecule on PtIr@GO cluster is depicted in Fig. 9.

Adsorption energy, enthalpy, and Gibbs free energy were calculated by using Eq. 1 in Supporting Information. Moreover, electrophilicity, chemical potential, electronegativity, chemical hardness, adsorption enthalpy, and HLG values were computed for both spin down and spin up molecular orbitals (β and α molecular orbitals respectively). The results given in Table 1 are optimized for PtIr@GO composites/DMAB system and were found using the HOMO/LOMO data^50,51^. In here, the values were computed by using Eqs 4–7 where ${\rm{I}}\cong -\,{\epsilon }_{{\rm{HOMO}}}$ and $A\cong -\,{\epsilon }_{{\rm{LOMO}}}$ for PtIr@GO composites/DMAB system. Additionally, the hemical potential value was calculated by using Eq. 5 where ${\rm{I}}\cong -\,{\epsilon }_{{\rm{HOMO}}}$ of free DMAB (donor) and $A\cong -\,{\epsilon }_{{\rm{LOMO}}}$ of the cluster (acceptor)^50–54^.

**Table 1:** HOMO/LUMO data, chemical hardness (*η*), the chemical potential (μ), electronegativity (*χ*), electrophilicity (ω), adsorption energy (∆E), adsorption enthalpy (∆H) and adsorption Gibbs free energy (∆G) and atomic distance values. (Units of energy and distance values are kcal/mol and Å, respectively)

Properties	PtIr@GO Cluster		PtIr@GO Cluster with adsorbed DMAB	
	α MO (Spin Up)	β MO (Spin down)	α MO (Spin Up)	β MO (Spin Down)
HOMO	−110.5	−114.5	−108.3	−111.3
LUMO	−69.4	−71.4	−62.2	−65.1
Chemical Hardness	48.2	47.2	23.1	23.1
Chemical Potential	−90.0	−92.9	−85.2	−88.2
Electronegativity	90.0	92.9	85.2	88.2
Electrophilicity	196.9	200.7	157.5	168.4
HLG	41.1	43.0	46.1	46.2
∆E	—	—	−33.4	
∆H	—	—	−34.0	
∆G	—	—	−21.6	
d(Pt-C)	2.059, 2.805		2.077, 2.775	
d(Ir-C)	2.110, 2.061		2.120, 2.089	
d(Ir-B)	—		2.299	

HOMO/LUMO data of α electrons and HOMO/LUMO data of β electrons for determined GO composition were calculated to be 124.3, 70.2 and 124.3,87.3 kcal/mol, respectively.

Before DMAB adsorption, chemical potential values for α and β molecular orbitals were calculated to be 117.7 and 118.7 respectively by using Eq. 5 where ${\rm{I}}\cong -\,{\epsilon }_{{\rm{HOMO}}}$ of free DMAB (donor) and $A\cong -{\epsilon }_{{\rm{LOMO}}}$ of the cluster (acceptor)^50–54^. These data indicate that PtIr@GO cluster has a large chemical potential value for interaction with DMAB molecule. The activity of PtIr@GO cluster according to electronegativity, chemical hardness electrophilicity, chemical potential and data have been determined.

It was found that the computed chemical potential value of the nanocluster was related to the energy of the adsorption. Therefore, the chemical potential for Nano Material was found to be low and the adsorption energy obtained for DMAB was found to be a low value^55,56^.

After DMAB adsorption on PtIr@GO cluster, electrophilicity, chemical potential, chemical hardness, electronegativity data have decreased while HLG value have increased. The relative adsorption energy value for DMAB adsorption was −33.4 kcal/mol on PtIr@GO cluster meaning DMAB was strongly adsorbed on it. Furthermore, Gibbs free energy (ΔG) for DMAB adsorption on the cluster was calculated to be −21.6 kcal/mol, which designates adsorption of DMAB molecule occurs simultaneously on the PtIr@GO cluster.

**Figure 10:**
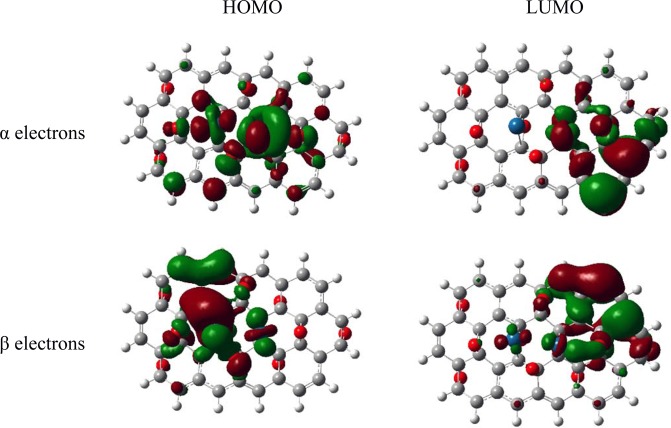
The HOMO/LUMO findings for α and β electrons in the optimized PtIr@GO cluster.

The HOMO-LUMO gap was extensively utilized as a pointer of kinetic stability, and it has been usually known that if the gap is small, the kinetic stability is low, and the chemical reactivity is high because it designates energetically favorable for the LUMO to gain electrons or for the HOMO to lose electrons. Based on the Table 1, it is clearly mentioned that the α-HLG gap value of 41.1 kcal/mol for the PtIr@GO cluster is significantly smaller than the α-HLG gap value of 54.1 kcal/mol for the GO cluster while β-HLG gap value of 43.0 kcal/mol for the PtIr@GO cluster is somewhat larger than the β -HLG gap value of 37.1 kcal/mol for the GO cluster. It can be also seen that the difference between α-HLG values of the PtIr@GO cluster and the GO cluster have been computed to be 13 kcal/mol and the difference between β-HLG values of the PtIr@GO cluster and the GO cluster is computed as 5.9 kcal/mol. The difference between α-HLG values is more than twice higher than the difference between β-HLG values. Based on this it has been concluded the chemical reactivity of the GO cluster has been considerably improved with the addition of Pt and Ir atoms on the cluster even if β-HLG gap value for the PtIr@GO cluster is somewhat larger than the β-HLG gap value for the GO cluster. In other words, the chemical reactivity of the PtIr@GO cluster is higher than that of GO cluster. The HOMO/LUMO results of α and β electrons for the PtIr@GO cluster are shown in Fig. 10. For the PtIr@GO cluster, it is shown that HOMOs of α and β electrons are localized on Pt and Ir atoms while LUMOs of α and β electrons are localized on only Ir atom. This explains that adsorption of DMAB on Ir atom is most favorable.

**Figure 11:**
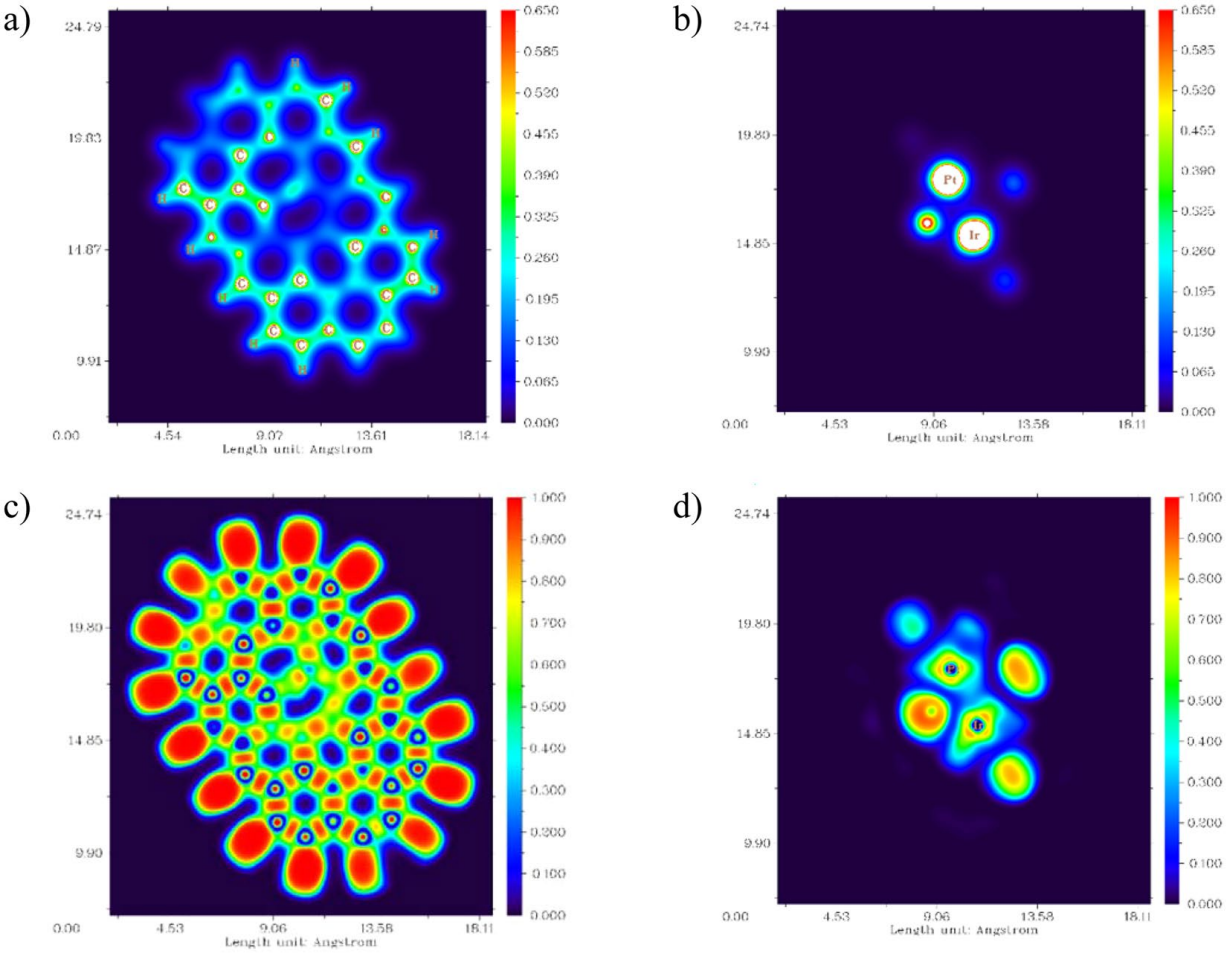
The electron density (ED) distribution map for optimized PtIr@GO cluster: (**a**) At the level of carbon atoms of the cluster and (**b**) At the level of Pt and Ir atoms of the cluster, The electronic localization function (ELF) distribution map for optimized PtIr@GO cluster: (**c**) At the level of carbon atoms of the cluster and (**d**) At the level of Pt and Ir atoms of the cluster.

**Figure 12:**
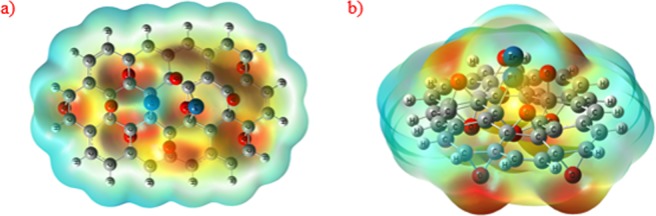
The electrostatic potential (ESP) distribution for optimized PtIr@GO cluster (**a**) Top view and (**b**) Side view.

In addition, parts a and b of Fig. 11 display the electron density (ED) distribution map for PtIr@GO cluster. The electron density distribution representations have pointed out that EDs are located mainly on the Pt and Ir. The electronic localization function (ELF) distribution map^57–60^ has been shown in part c and d of Fig. 11 for PtIr@GO cluster. ELF is a valued tool for determining the position of the pairs of electron^60^. The ELF function helps to understand the empirical concept of the localization of electron, in particular the location of the paired electron. According to the ELF graph, the atoms where they showed larger values of the ELF in PtIr@GO cluster are Pt and Ir atoms. Besides, the negative and positive regions electrostatic potential (ESP) distribution (shown in Fig. 12) on the van der Waals surface were represented by the red and blue colors, respectively^61,62^. The ESP distribution’s analysis of PtIr@GO cluster result shows that blue color regions (positiveregion) are localized on Pt and Ir atoms (mostly Ir atoms). This ESP distrubition is reliable with the charge distribution achieved by the analysis of Mulliken population. The charges values for Pt and Ir atoms are +0.072 and +0.323, respectively. It should also be noted that some positive (blue) regions of PtIr@GO cluster are positioned round the ending hydrogen atoms surrounding the PtIr@GO cluster. Nonetheless, it is inappropriate to consider these blue regions because these hydrogen atoms have been used to saturate the carbon atoms.

## Conclusions

Herein, the preparation, characterization of Composites of Platinum-Iridium Alloy Nanoparticles and Graphene Oxide, their catalytic activities and activation parameters were investigated for dehydrogenation of DMAB. The complete conversion of DMAB was obtained at ambient conditions. Some advanced process including TEM, HRTEM, XRD, Raman, and XPS, etc. were used to characterize PtIr@GO nanoparticles. The prepared platinum-iridium nanoparticles were determined to be highly active, reusable and durable for the catalytic dehydrogenation of DMAB. The reaction rate of catalytic reaction of DMAB include PtIr@GO nanoparticles was found to be −d [DMAB]/dt = +d[H_2_]/dt = k_*obs*_ [PtIr@GO]^1.0721^ [DMAB]^0.6621^. The activation parameters such as activation energy (Ea: 19.06 ± 2 kJ mol^−1^), entropy (ΔS: −178.18 ± 2 J mol^−1^ K^−1^) and enthalpy (ΔH: 16.53 ± 2 kJ mol^−1^) were obtained using Arrhenius and Eyring equations. Initial TOF value of PtIr@GO was calculated to be 225.64 h^−1^ for dehydrogenation of DMAB at 298 K, and this value is very high compared to the previous studies in literature as shown in Table S1. In short, PtIr@GO nanoparticles can be used as an effective catalyst for hydrogen storage materials. Theoretical results based on HOMO/LUMO, electronegativity, chemical potential, chemical hardness, adsorption energy, adsorption enthalpy (∆H) and adsorption Gibbs free energy (∆G) values, as well as ED, ELF, ESP distributions, support the experimental result which is accordingly high activity of PtIr@GO catalyst for dehydrogenation of DMAB. As a result of the experimental and calculations obtained, it was found that synthesized PtIr@GO nanocatalyst has high catalytic activity, stability, and reusable properties. With these results, it is concluded that this catalyst can be used for the release of hydrogen in the DMAB as a hydrogen source.

## Supplementary information

Supplementary Information
