# Supplementary material for: Composites of Platinum-Iridium Alloy Nanoparticles and Graphene Oxide for the Dimethyl Amine Borane (DMAB) dehydrogenation at ambient conditions: An Experimental and Density Functional Theory Study
Source: Sci Rep. 2019 Oct 29;9:15543. doi: 10.1038/s41598-019-52038-3 (PMC6820564; doi:10.1038/s41598-019-52038-3)
Supplement: Supplementary file 1 — Supplementary Information [file 41598_2019_52038_MOESM1_ESM.docx]

**SUPPORTING INFORMATION**

**Composites of Platinum-Iridium Alloy Nanoparticles and Graphene Oxide for the Dimethyl Amine Borane (DMAB) dehydrogenation at ambient conditions: An Experimental and Density Functional Theory Study**

Betül Sen^1^, Ayşenur Aygun^1^, Aysun Şavk^1^, Mehmet Harbi Çalımlı^1,2^, Mehmet Ferdi Fellah^3,^ Fatih Sen^1^*

^1^Sen Research Group, Biochemistry Department, Faculty of Arts and Science, Dumlupınar University, Evliya Çelebi Campus, 43100 Kütahya, Turkey. E-mail: fatihsen1980@gmail.com

^2^Tuzluca Vocational High School, Igdir University, Igdir, Turkey

^3^Department of Chemical Engineering, Bursa Technical University, Mimar Sinan Campus, 16310, Bursa, Turkey

**Materials**

The water used in all experiments was prepared by a Millipore water purification system. Tetrahydrofuran (THF, Merck, 99.5%) was prepared under argon atmosphere. GO (graphene oxide), lithium triethyl borohydride, dimethylamine-borane, ethanol were purchased from Sigma Aldrich. PtCl_4_–IrCl_3_ (99%) salts were purchased from Alfa Aesar.

**Instrumentation**

TEM and HRTEM analysis of PtIr@GO alloy nanoparticles were performed using JEOL 200 kV TEM microscope. X-ray diffraction (XRD) was performed using a Panalytical Empyrean diffractometer with Ultima + theta-theta high resolution goniometer, the X-ray generator (Cu K radiation, λ = 1.54056Å) with an operation conditions at 45 kV and 40 mA. A Specs spectrometer was used for X-ray photoelectron spectroscopy (XPS) measurements using K lines of Mg (1253.6 eV, 10 mA) as an X-ray source.


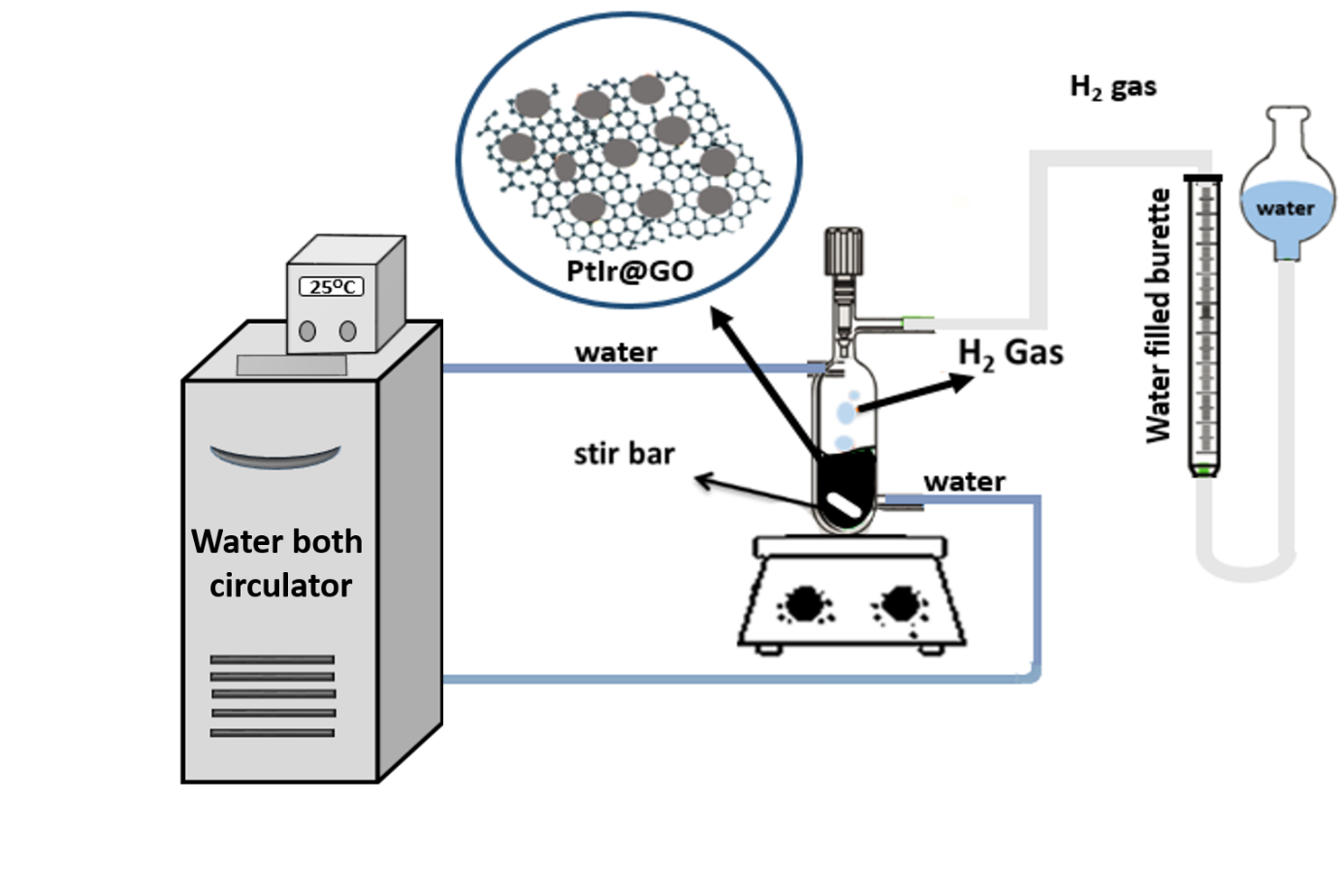


**Fig. S1.** The reaction set up for the dehydrocoupling of DMAB with the help of prepared catalyst.

**Computational Method**

The convergence criteria are 12x10^-4^ for gradients of root-mean-square (rms) displacement, 18x10^-4^ for max displacement, 3x10^-4^ for rms force and 45x10^-5^ for max force for theoretical calculations utilized in this study. The theoretical methodology utilized here: Firstly, the Spin Multiplicity (SM) for the system including adsorbing molecule and the cluster has been determined by SPE calculations. SPEs were calculated for different numbers of SM for separately system, and then the number of SM which gives the lowest energy based on SPE calculation was accepted to be final SM number for the related system. Then, the adsorbing molecule (DMAB molecule here) and the cluster were structurally optimized by EG calculations. Following equation has been used in order to compute the relative energy values for calculations.

$\Delta\left( E/H \right)= \left( E/H \right)_{System}-\left( E/H \right)_{Adsortive}-{(E/H)}_{Cluster}$ (1)

Here, (E/H)_System_ is the calculated energy for the optimized system which contains the geometries of the adsorbing molecule and the cluster, (E/H)_Adsorbtive_ is the calculated energy for the adsorbing molecule, e.g. DMAB molecule and (E/H)_Cluster_ is the calculated energy for the original cluster.

|  |
| --- |
| 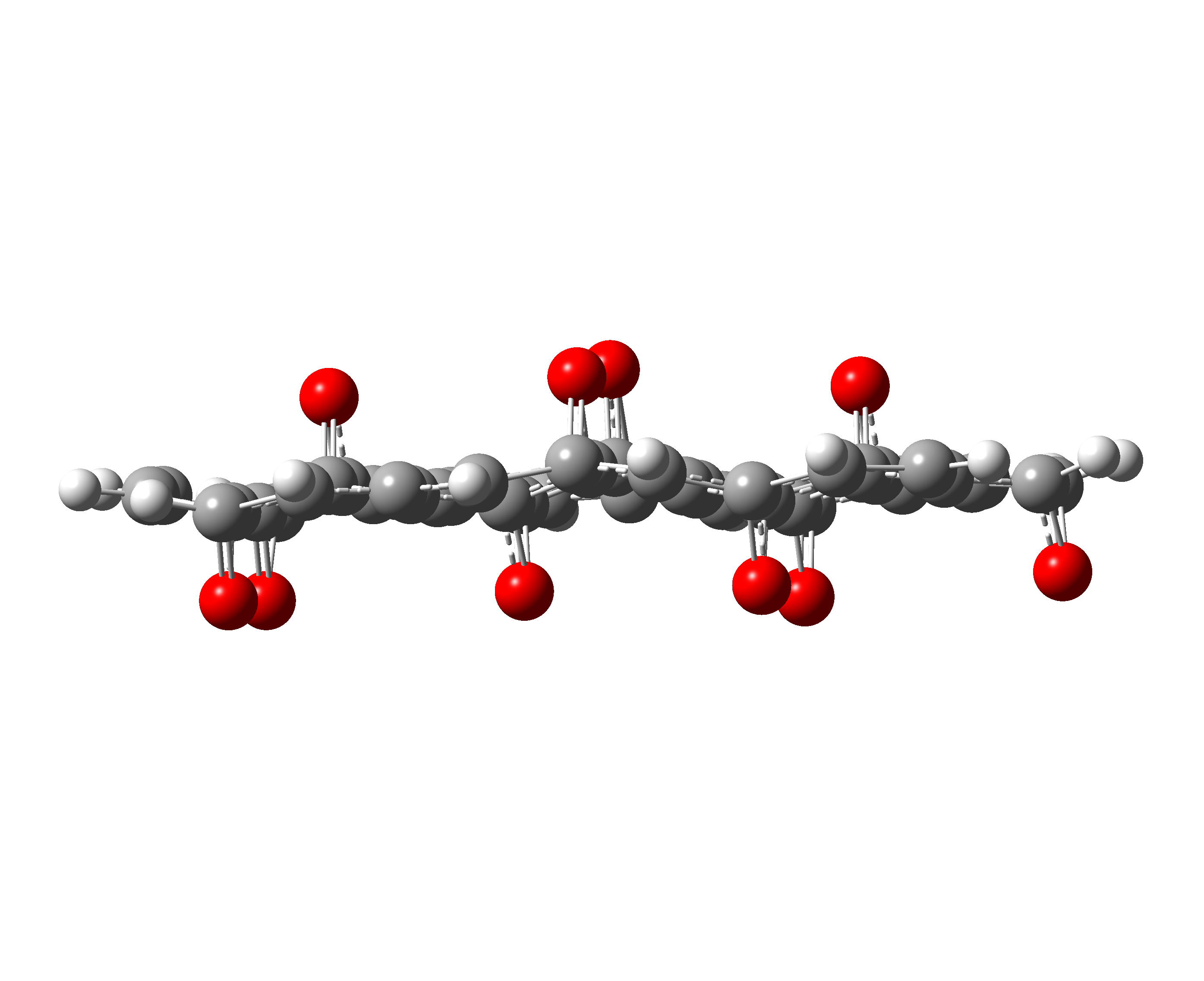 |
| **Fig. S2.** The optimized structure of GO cluster with top view and side view (p1 and p2 represent the possible position for the location of Pt and Ir atom.  ****  **Fig. S3.** The C1s XPS spectra of PtIr@GO alloy nanoparticles.  Fig. S3 shows C 1s core level XPS spectra of PtIr@GO alloy nanoparticles. A peak is located at about 284.2 eV in the C1s spectrum attributed to the sp^2^ carbon of graphite. The carbon backbone of the GO is also active in the presence of this peak. Species with binding energies at about 285.6, 286.8 and 289.0 eV are related with the presence of sp^3^ carbon, C-O and C=O (O=C-O), respectively and these may be connected with environment and adsorbed oxygenated species. |


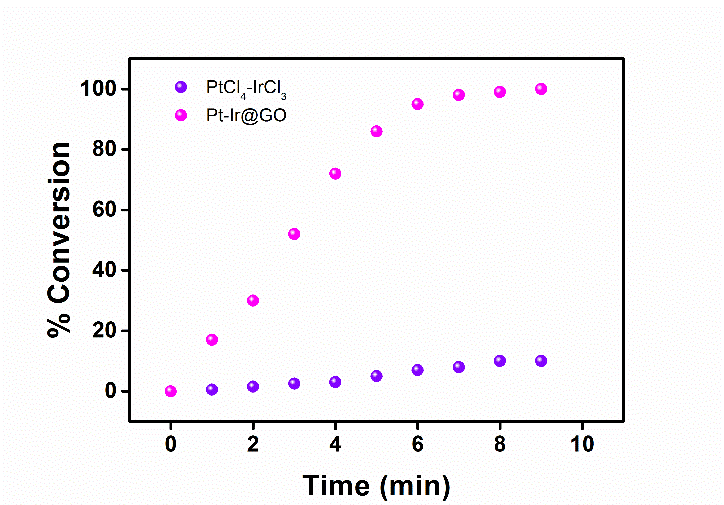


**Fig. S4.** Percent conversion versus time graph with 7.5 % mol of PtIr@GO and PtCl_4_-IrCl_3_ (precursor materials of the prepared catalyst).


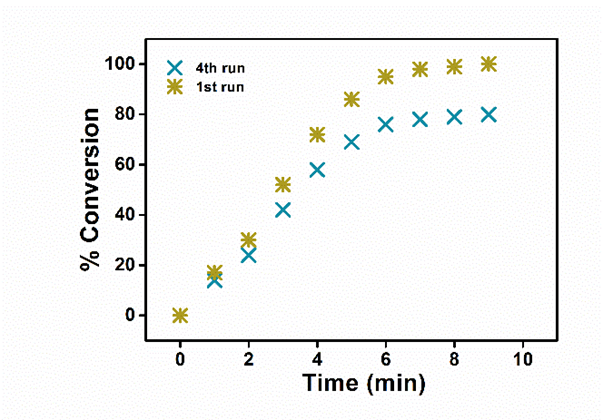


**Fig. S5.** Percent conversion versus time graph for reusability experiments of PtIr@GO nanoparticles for dehydrogenation of DMAB.


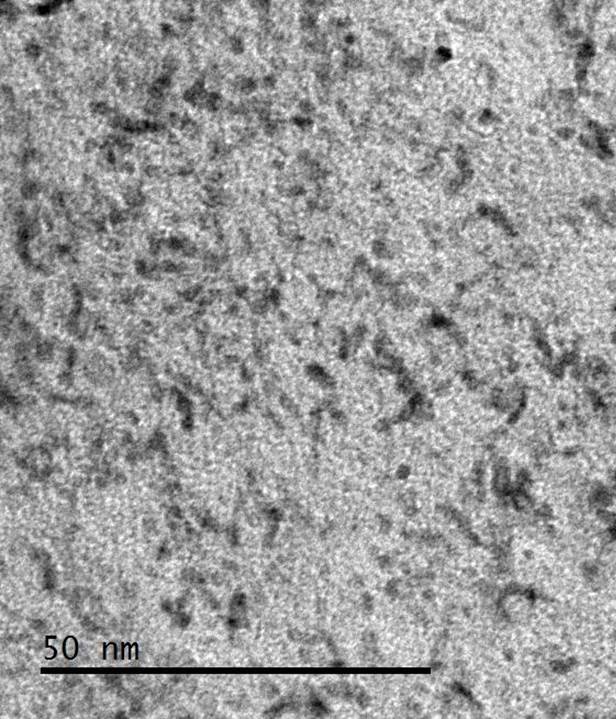


**Fig. S6.** Transmission Electron Microscopy image of prepared nanoparticles after reusability experiments.

**Table S1.** The comparison of TOF values of prepared catalysts in literature.

| Entry | (Pre) Catalysts | Conv. (%) | TOF | Ref |
| --- | --- | --- | --- | --- |
| 1 | **Graphene Oxide based binary Platinum-Iridium Nanomaterials** | **100** | **225.64** | **This study** |
|  | **Graphene Oxide based Platinum Nanomaterials** | **100** | **35.82** |  |
|  | **Graphene Oxide based Iridium Nanomaterials** | **100** | **29.74** |  |
| 2 | Rhodium (III) chloride | 90 | 7.9 | ^1^ |
| 3 | Carbon Stabilized Palladium particles | 95 | 2.8 | ^1^ |
| 4 | Trans ruthenium based complex | 100 | 12.4 | ^1^ |
| 5 | Titanium based complex | 100 | 12.3 | ^2^ |
| 6 | Iridium complex | 95 | 0.7 | ^1^ |
| 7 | Rhodium based complex | 95 | 1.7 | ^1^ |
| 8 | Rhodium Complexes | 100 | 12.5 | ^1^ |
| 9 | Rhodium based complex | 95 | 12.0 | ^1^ |
| 10 | Ruthenium based complex | 100 | 1.5 | ^3^ |
| 11 | Iridium (III) chloride | 25 | 0.3 | ^1^ |
| 12 | Chromium based complex | 97 | 13.4 | ^4^ |
| 13 | Rhodium based complex | 90 | 8.2 | ^1^ |
| 14 | Rhodium based complex | 100 | 2.6 | ^5^ |
| 15 | Rhodium based complex | 100 | 4.3 | ^1^ |
| 16 | Copper based complex | 100 | 0.3 | ^6^ |
| 17 | Nickel based complex | 100 | 3.2 | ^7^ |
| 18 | Rhodium based complex | 100 | 0.9 | ^8^ |
| 19 | Chromium based complex | 97 | 19.9 | ^4^ |
| 20 | Ruthenium based complex | 40 | 1.6 | ^1^ |
| 21 | Ruthenium (III) chloride | 77 | 2.7 | ^1^ |
| 22 | Titanium based complex | 100 | 420.0 | ^9^ |
| 23 | Ruthenium based complex | 70 | 2.5 | ^1^ |
| 24 | Rhodium based complex | 5 | 0.1 | ^1^ |
| 25 | Trans Palladium based complex | 20 | 0.2 | ^1^ |
| 26 | BA based Platinum nanomaterials | 100 | 24.88 | ^10^ |
| 27 | TBA based Platinum nanoparticles | 100 | 31.24 | ^10^ |
| 28 | Activated Carbon Stabilized Pt Nanoparticles | 100 | 15.0 | ^11^ |
| 29 | Activated Carbon Stabilized Pt Nanoparticles | 100 | 34.14 | ^12^ |
| 30 | PVP stabilized Palladium-Cobalt nanoparticles | 100 | 330 | ^13^ |
| 31 | Ruthenium based trimetallic nanomaterials | 100 | 727 | ^14^ |
| 32 | Carbon-nanotube Based Ruthenium-cobalt nanoparticles | 100 | 775.28 | ^15^ |
| 33 | PEDOT supported Palladium Nickel Nanoparticles | 100 | 451.28 | ^16^ |
| 34 | Polymer-graphene based Platinum Nanomaterials | 100 | 42.94 | ^17^ |
| 35 | Graphene oxide stabilized Palladium-Nickel Nanomaterials | 100 | 271.90 | ^18^ |
| 36 | Graphene oxide based Palladium Nanoparticles | 100 | 38.02 | ^19^ |
| 37 | Carbon black hybrid supported platinum nanomaterials | 100 | 70.28 | ^20^ |
| 38 | Polymer-supported Ruthenium-Nickel Nanoparticles | 100 | 458.57 | ^21^ |

**References**

S1. Chandra, M. & Xu, Q. A high-performance hydrogen generation system: Transition metal-catalyzed dissociation and hydrolysis of ammonia–borane. *J. Power Sources* **156**, 190–194 (2006).

S2. Sloan, M. E. *et al.* Homogeneous Catalytic Dehydrocoupling/Dehydrogenation of Amine−Borane Adducts by Early Transition Metal, Group 4 Metallocene Complexes. *J. Am. Chem. Soc.* **132**, 3831–3841 (2010).

S3. Friedrich, A., Drees, M. & Schneider, S. Ruthenium-Catalyzed Dimethylamineborane Dehydrogenation: Stepwise Metal-Centered Dehydrocyclization. *Chem. - A Eur. J.* **15**, 10339–10342 (2009).

S4. Kawano, Y. *et al.* Dehydrocoupling Reactions of Borane−Secondary and −Primary Amine Adducts Catalyzed by Group-6 Carbonyl Complexes: Formation of Aminoboranes and Borazines. *J. Am. Chem. Soc.* **131**, 14946–14957 (2009).

S5. Sloan, M. E., Clark, T. J. & Manners, I. Homogeneous Catalytic Dehydrogenation/Dehydrocoupling of Amine-Borane Adducts by the Rh(I) Wilkinson’s Complex Analogue RhCl(PHCy _2_ ) _3_ (Cy = cyclohexyl). *Inorg. Chem.* **48**, 2429–2435 (2009).

S6. Keaton, R. J., Blacquiere, J. M. & Baker, R. T. Base metal catalyzed dehydrogenation of ammonia-borane for chemical hydrogen storage. *J. Am. Chem. Soc.* **129**, 1844–1845 (2007).

S7. Robertson, A. P. M., Suter, R., Chabanne, L., Whittell, G. R. & Manners, I. Heterogeneous dehydrocoupling of amine-borane adducts by skeletal nickel catalysts. *Inorg. Chem.* **50**, 12680–12691 (2011).

S8. Cory A. Jaska *et al.* Transition metal-catalyzed formation of boron-nitrogen bonds: Catalytic dehydrocoupling of amine-borane adducts to form aminoboranes and borazines. *J. Am. Chem. Soc.* **125**, 9424–9434 (2003).

S9. Li, Y. *et al.* Polymeric Micelle Assembly for the Smart Synthesis of Mesoporous Platinum Nanospheres with Tunable Pore Sizes. *Angew. Chemie Int. Ed.* **54**, 11073–11077 (2015).

S10. Erken, E. *et al.* New Pt(0) Nanoparticles as Highly Active and Reusable Catalysts in the C1–C3 Alcohol Oxidation and the Room Temperature Dehydrocoupling of Dimethylamine-Borane (DMAB). *J. Clust. Sci.* **27**, 9–23 (2016).

S11. Sen, F., Karatas, Y., Gulcan, M. & Zahmakiran, M. Amylamine stabilized platinum(0) nanoparticles: active and reusable nanocatalyst in the room temperature dehydrogenation of dimethylamine-borane. *RSC Adv.* **4**, 1526–1531 (2014).

S12. Li, C., Sato, T. & Yamauchi, Y. No Title. **52**, 8050–8053 (2013).

S13. Çelik, B. *et al.* Monodispersed palladium–cobalt alloy nanoparticles assembled on poly(N-vinyl-pyrrolidone) (PVP) as a highly effective catalyst for dimethylamine borane (DMAB) dehydrocoupling. *RSC Adv.* **6**, 24097–24102 (2016).

S14. Sen, B., Kuzu, S., Demir, E., Onal Okyay, T. & Sen, F. Hydrogen liberation from the dehydrocoupling of dimethylamine–borane at room temperature by using novel and highly monodispersed RuPtNi nanocatalysts decorated with graphene oxide. *Int. J. Hydrogen Energy* **42**, 23299–23306 (2017).

S15. Şen, B., Kuzu, S., Demir, E. & Akocak, SüleymanŞSen, F. Highly Monodisperse RuCo Nanoparticles Decorated on Functionalized Multiwalled Carbon Nanotube with The Highest Observed Catalytic Activity in The Dehydrogenation of Dimethylamine−borane. *Int. J. Hydrogen Energy* **42**, 23292–23298 (2017).

S16. Sen, B., Kuzu, S., Demir, E., Yıldırır, E. & Sen, F. Highly efficient catalytic dehydrogenation of dimethyl ammonia borane via monodisperse palladium–nickel alloy nanoparticles assembled on PEDOT. *Int. J. Hydrogen Energy* **42**, 23307–23314 (2017).

S17. Sen, B., Kuzu, S., Demir, E., Akocak, S. & Sen, F. Polymer-graphene hybride decorated Pt nanoparticles as highly efficient and reusable catalyst for the dehydrogenation of dimethylamine–borane at room temperature. *Int. J. Hydrogen Energy* **42**, 23284–23291 (2017).

S18. Sen, B., Kuzu, S., Demir, E., Akocak, S. & Sen, F. Monodisperse palladium–nickel alloy nanoparticles assembled on graphene oxide with the high catalytic activity and reusability in the dehydrogenation of dimethylamine–borane. *Int. J. Hydrogen Energy* **42**, 23276–23283 (2017).

S19. Şen, B. *et al.* Monodisperse palladium nanoparticles assembled on graphene oxide with the high catalytic activity and reusability in the dehydrogenation of dimethylamine-borane. *Int. J. Hydrogen Energy* **43**, 20176–20182 (2018).

S20. Sen, B., Şavk, A. & Sen, F. Highly efficient monodisperse Pt nanoparticles confined in the carbon black hybrid material for hydrogen liberation. *J. Colloid Interface Sci.* **520**, 112–118 (2018).

S21. Sen, B., Kuyuldar, E., Demirkan, B., … T. O.-J. of colloid and & 2018, undefined. Highly efficient polymer supported monodisperse ruthenium-nickel nanocomposites for dehydrocoupling of dimethylamine borane. *Elsevier*
